# Supplementary material for: Fibroblast-specific PRMT5 deficiency suppresses cardiac fibrosis and left ventricular dysfunction in male mice
Source: Nat Commun. 2024 Mar 19;15:2472. doi: 10.1038/s41467-024-46711-z (PMC10951424; doi:10.1038/s41467-024-46711-z)
Supplement: Supplementary file 1 — Supplementary Information [file 41467_2024_46711_MOESM1_ESM.pdf]

a

【$Postn^{MCM}$; $Prmt5^{fl/fl}$】

sex	No. of live mice	Genotype of live mice	
		$Prmt5^{fl/fl}$	$Postn^{MCM}$; $Prmt5^{fl/fl}$
♂	123	76	47
		33.6%	20.8%
♀	103	51	52
		22.6%	23.0%

sex	No. of live mice	Genotype of live mice	
		$Prmt5^{fl/fl}$	$Postn^{MCM}$; $Prmt5^{fl/fl}$
♂♀	226	127	99
		56.2%	43.8%

b

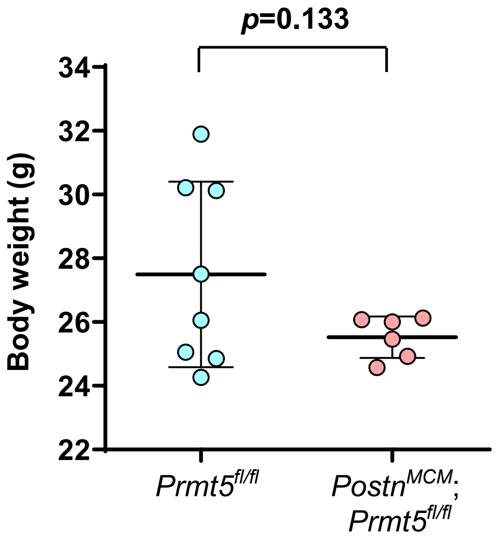

Supplementary Figure 1

$Postn^{MCM}$;$Prmt5^{fl/fl}$ mice were born in an expected Mendelian ratio (a). The body weight of these mice was not significantly different from that of $Prmt5^{fl/fl}$ mice (b). Values are presented as mean ± SD. n = 8 $Prmt5^{fl/fl}$ mice and 6 $Postn^{MCM}$;$Prmt5^{fl/fl}$ mice. Unpaired Student's *t*-test.

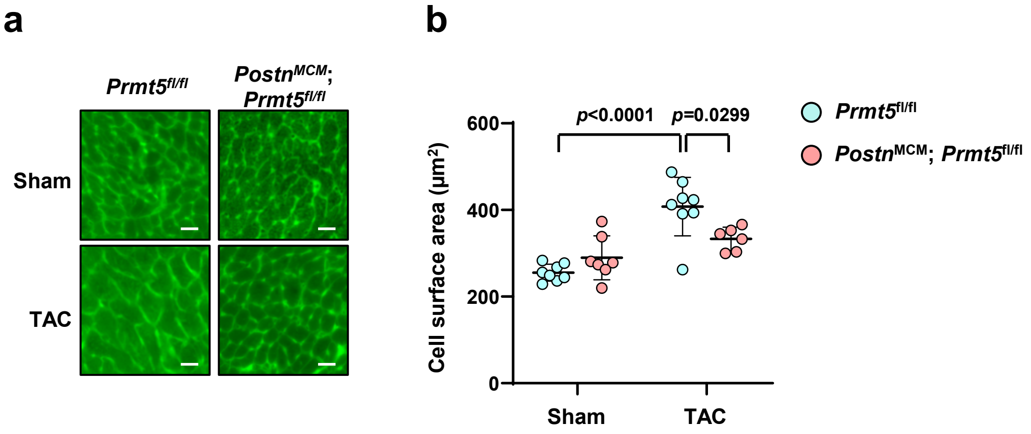

Supplementary Figure 2

Prmt5 knockout in *Postn*-positive fibroblasts suppresses cardiac hypertrophy induced by pressure overload. Representative images of heart tissue are shown. The surface of the cardiomyocytes was stained with FITC-labeled wheat germ agglutinin and their area was determined with ImageJ software (a). Values are presented as mean ± SD (n = 8 $Prmt5^{fl/fl}$ mice [sham], 7 $Postn^{MCM}$;$Prmt5^{fl/fl}$ mice [sham], 8 $Prmt5^{fl/fl}$ mice [TAC], and 6 $Postn^{MCM}$;$Prmt5^{fl/fl}$ mice [TAC]). (b). Two-way ANOVA, followed by Tukey's multiple comparison test.

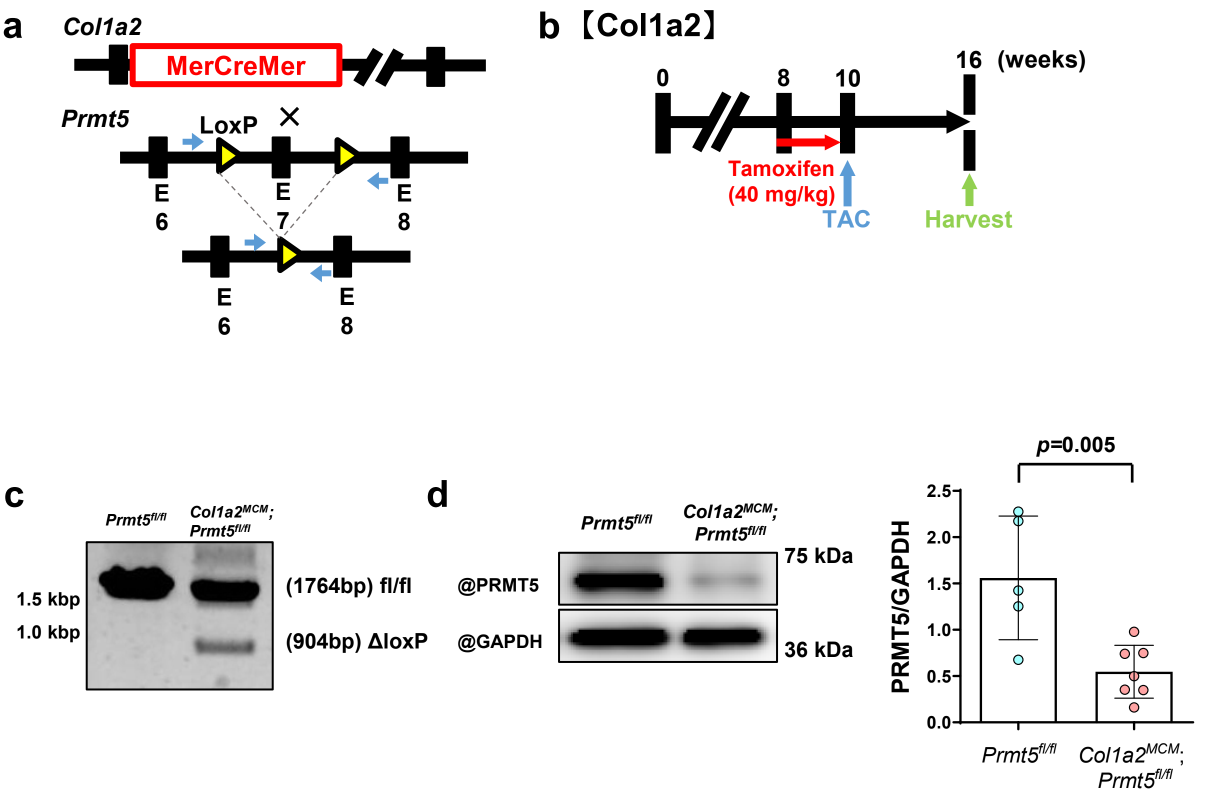

e

【*Col1a2*^{MCM}; *Prmt5*$^{fl/fl}$】

sex	No. of live mice	Genotype of live mice	
		Prmt5$^{fl/fl}$	*Col1a2*^{MCM}; *Prmt5*$^{fl/fl}$
♂	164	77	87
		23.2%	26.2%
♀	168	95	73
		28.6%	22.0%

sex	No. of live mice	Genotype of live mice	
		Prmt5$^{fl/fl}$	*Col1a2*^{MCM}; *Prmt5*$^{fl/fl}$
♂♀	332	172	160
		51.8%	48.2%

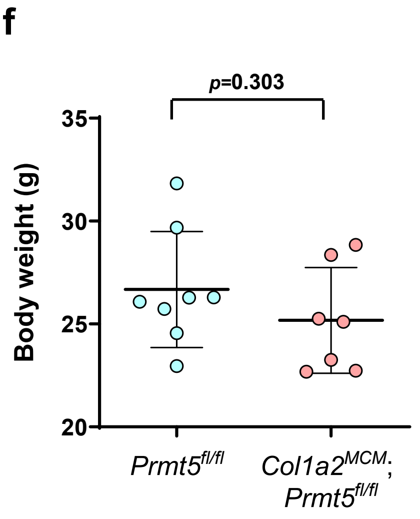

Supplementary Figure 3

Knockout of *Prmt5* in the fibroblasts of *Col1a2*^{MCM};*Prmt5*$^{fl/fl}$ mice was confirmed by western blotting. Fibroblasts from tamoxifen-treated mice were cultured, and their genome DNA and protein expression was determined (a-d). Values are presented as mean ± SD (n = 5 *Prmt5* mice and 7 *Col1a2*^{MCM};*Prmt5*$^{fl/fl}$ mice) (d). *Prmt5* knockout in *Col1a2*-positive fibroblasts did not cause significant changes in the growth of the mice. The mice were born in an expected Mendelian ratio (e). The body weight of these mice was not significantly different from that of *Prmt5*$^{fl/fl}$ mice (f). Values are presented as mean ± SD (n = 8 *Prmt5* mice and 7 *Col1a2*^{MCM};*Prmt5*$^{fl/fl}$ mice). Unpaired Student's *t*-test.

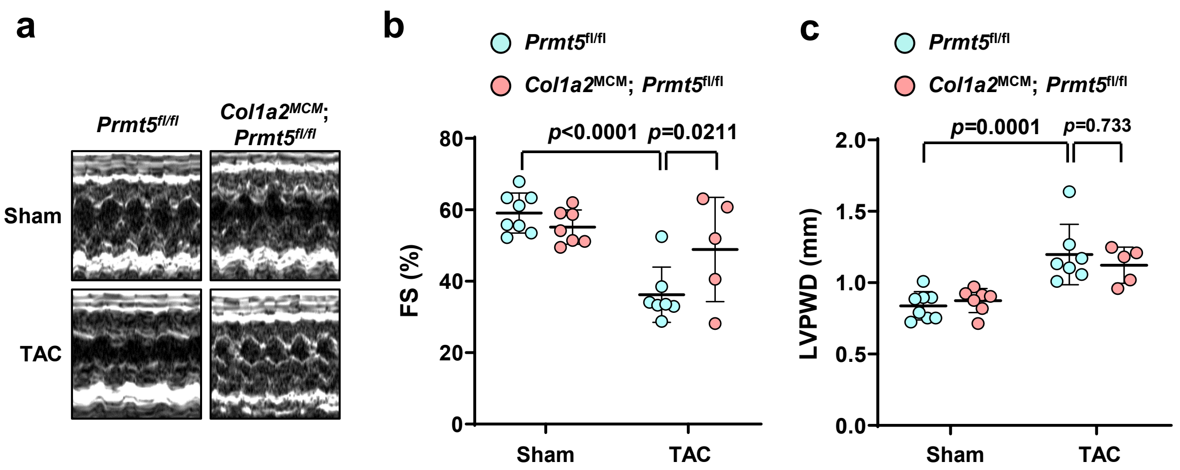

Supplementary Figure 4

Prmt5 knockout in fibroblasts suppresses cardiac dysfunction induced by pressure overload. *Prmt5* deficiency in fibroblast lineages attenuates pressure overload-induced cardiac dysfunction. Echocardiography analysis was performed on *Col1a2^{MCM};Prmt5$^{fl/fl}$* and *Prmt5$^{fl/fl}$* mice at 8 weeks after TAC surgery (a). Fractional shorting (b) and LVPDW (c) were calculated. Values are presented as mean ± (n = 8 *Prmt5$^{fl/fl}$* mice [sham], 7 *Col1a2^{MCM};Prmt5$^{fl/fl}$* mice [sham], 7 *Prmt5$^{fl/fl}$* mice [TAC], and 5 *Col1a2^{MCM};Prmt5$^{fl/fl}$* mice [TAC]). Two-way ANOVA, followed by Tukey's multiple comparison test.

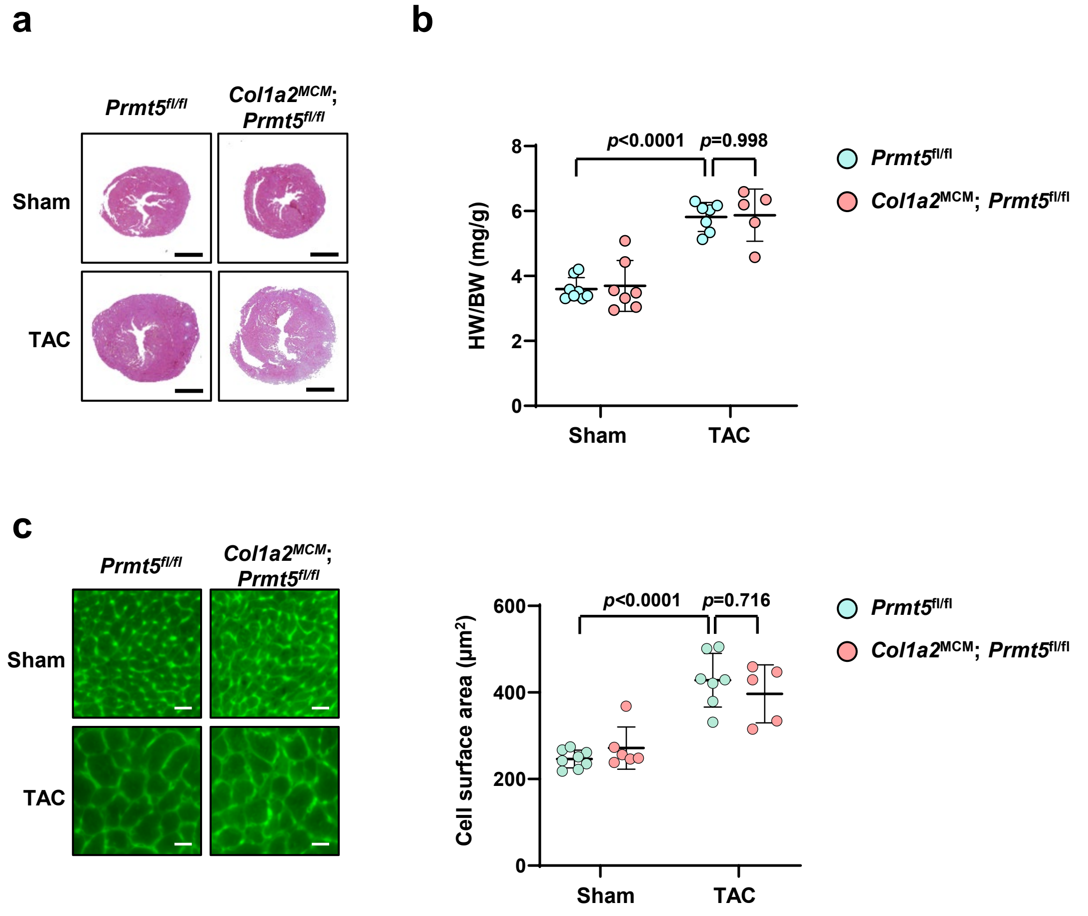

Supplementary Figure 5

Prmt5 knockout in *Col1a2*-positive fibroblasts suppresses cardiac hypertrophy induced by pressure overload. Representative images of heart tissue are shown. Heart-weight-to-body-weight ratio was calculated 8 weeks after TAC surgery (a). The surface of the cardiomyocytes was stained with FITC-labeled wheat germ agglutinin and their area was determined with ImageJ software (b). Values are presented as mean ± SD (n = 8 *Prmt5$^{fl/fl}$* mice [sham], 7 *Col1a2^{MCM};Prmt5$^{fl/fl}$* mice [sham], 7 *Prmt5$^{fl/fl}$* mice [TAC], and 5 *Col1a2^{MCM};Prmt5$^{fl/fl}$* mice [TAC]). Representative images of heart tissue are shown. The surface of the cardiomyocytes was stained with FITC-labeled wheat germ agglutinin and their area was determined with ImageJ software (c). Values are presented as mean ± SD (n = 8 *Prmt5$^{fl/fl}$* mice [sham], 6 *Col1a2^{MCM};Prmt5$^{fl/fl}$* mice [sham], 7 *Prmt5$^{fl/fl}$* mice [TAC], and 5 *Col1a2^{MCM};Prmt5$^{fl/fl}$* mice [TAC]). Two-way ANOVA, followed by Tukey's multiple comparison test.

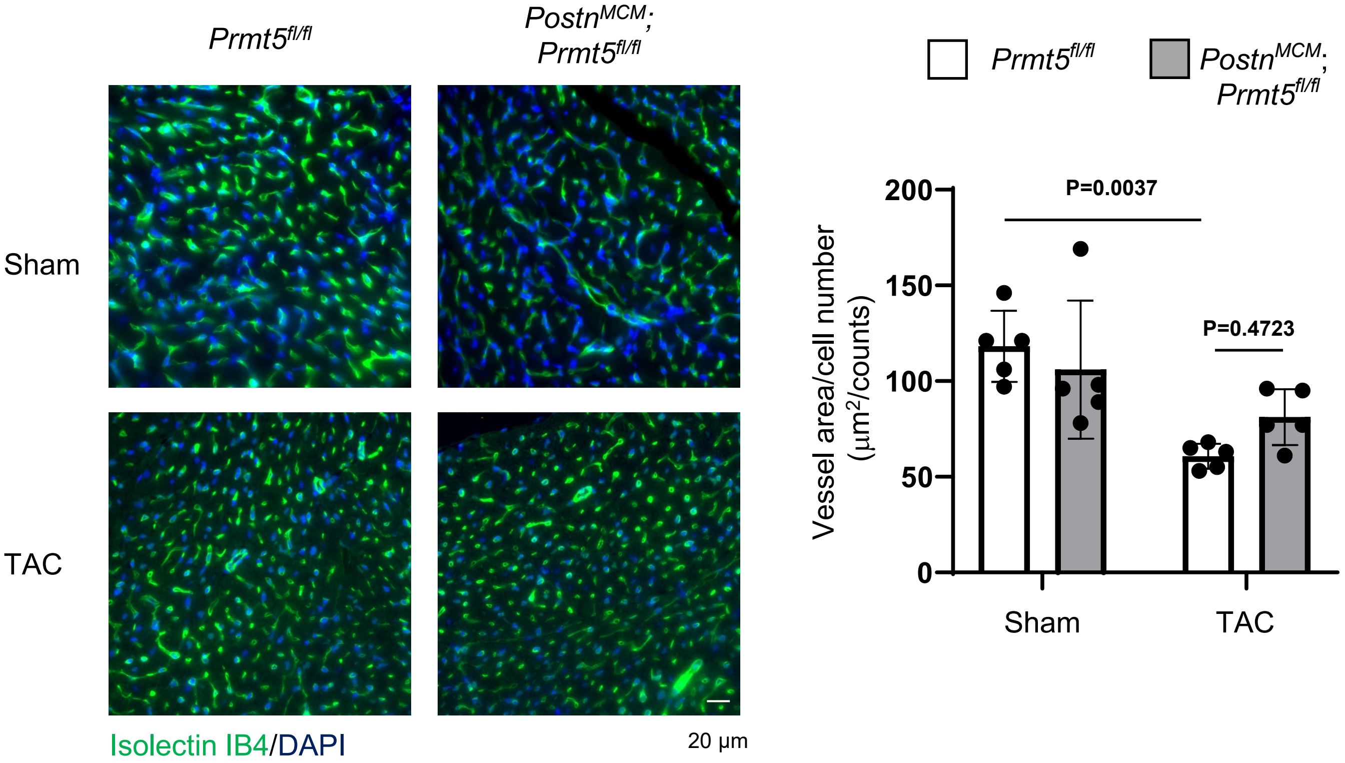

Supplementary Figure 6

Prmt5 knockout in *Postn*-positive fibroblasts did not change the cardiac capillary density after pressure overload. Representative images of blood vessels (green, Isolectin IB4) and nucleus (blue) in heart tissues are shown. Vessel density was measured with ImageJ software. Values are presented as mean ± SD (n = 5 mice/group). Two-way ANOVA, followed by Tukey's multiple comparison test.

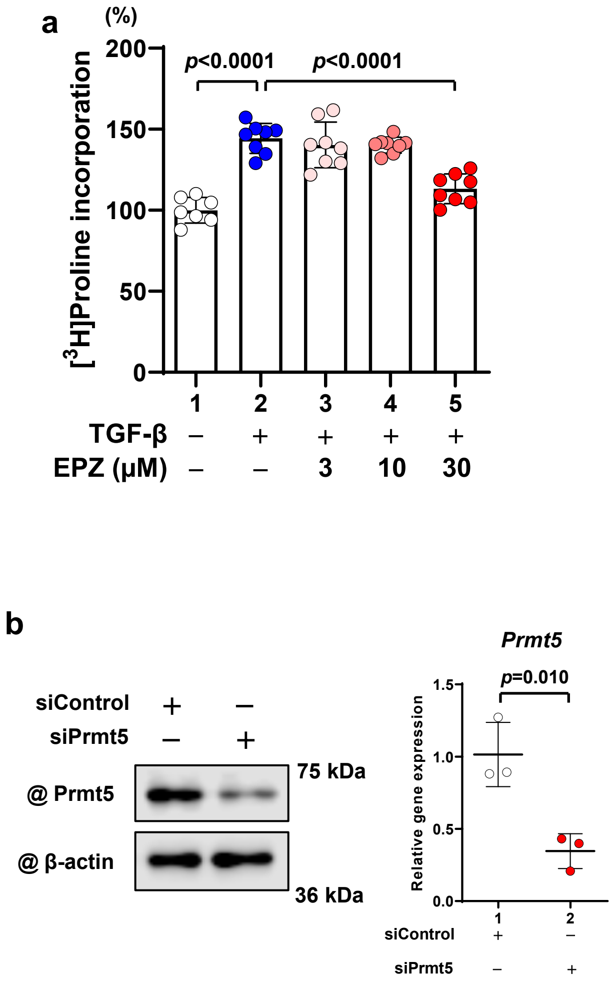

Supplementary Figure 7

Primary cultured cardiac fibroblasts from neonatal rats were treated with the PRMT5 inhibitor EPZ015666 at the indicated concentration and then stimulated with or without TGF-β (10 ng/mL). [^{3}H]-labeled L-proline uptake by the cells was measured after incubation for 48 h. Values are presented as mean ± SD. n=8 biological independent samples. One-way ANOVA, followed by Dunnett's multiple comparison test versus TGF-β treated group (a). *Prmt5* knockdown was confirmed in primary cultured cardiac fibroblasts by western blotting and quantitative PCR (b). Values are presented as mean ± SD. n=3 biological independent samples. Unpaired Student's *t*-test.

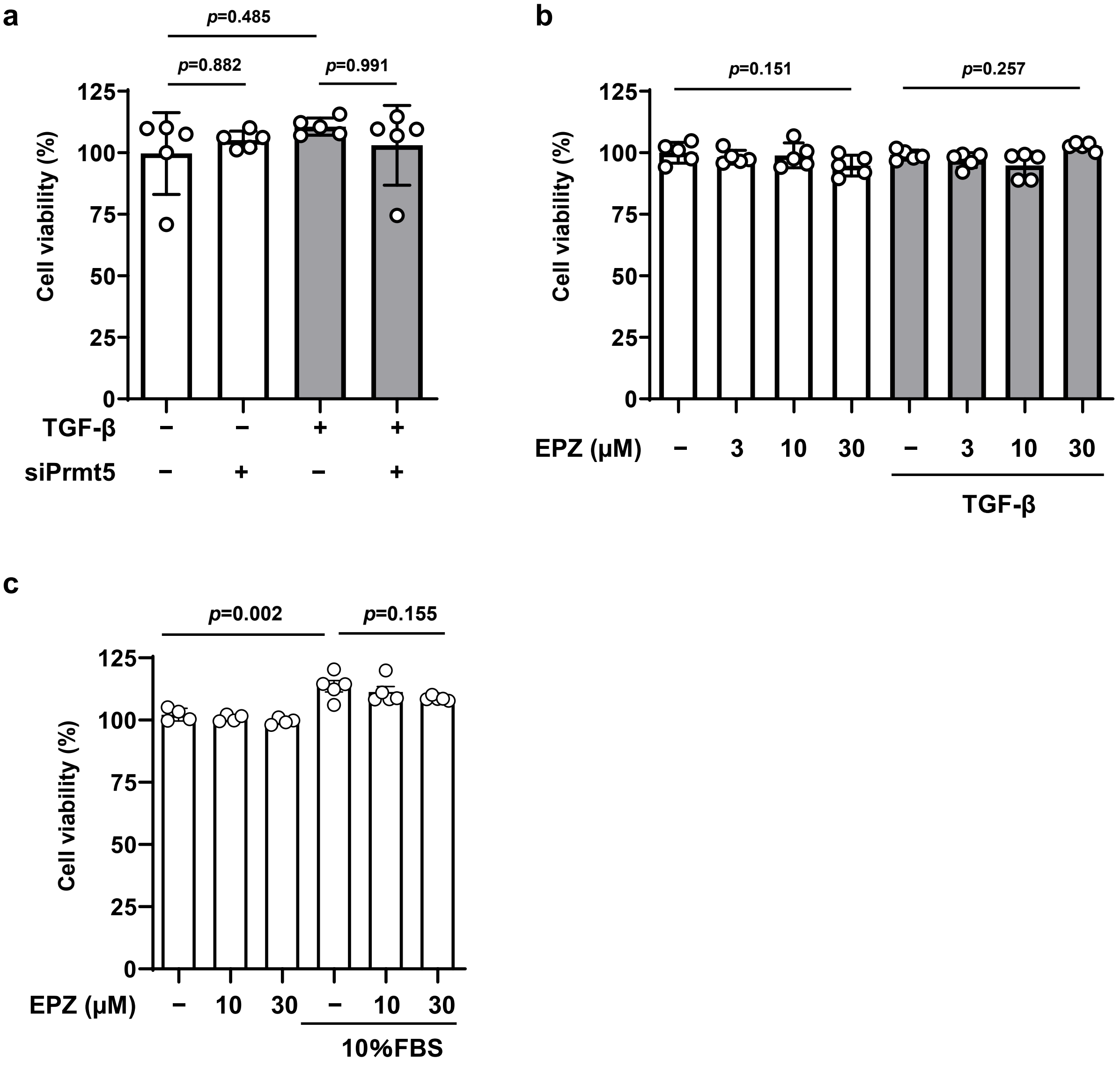

Supplementary Figure 8

(a) Primary cultured cardiac fibroblasts from neonatal rats were transfected with siRNA (siControl and siPrmt5) and then stimulated with or without TGF-β. (b and c) The cells were treated with EPZ015666 at the indicated concentration and then stimulated with or without TGF-β (10 ng/mL) (b) or 10%FBS (c). Cell viability was measured with cell counting kit-8 after incubation for 48 h. Values are presented as mean ± SD. n=5 biological independent samples. One-way ANOVA, followed by Dunnett's multiple-comparisons test or Tukey's multiple comparison test.

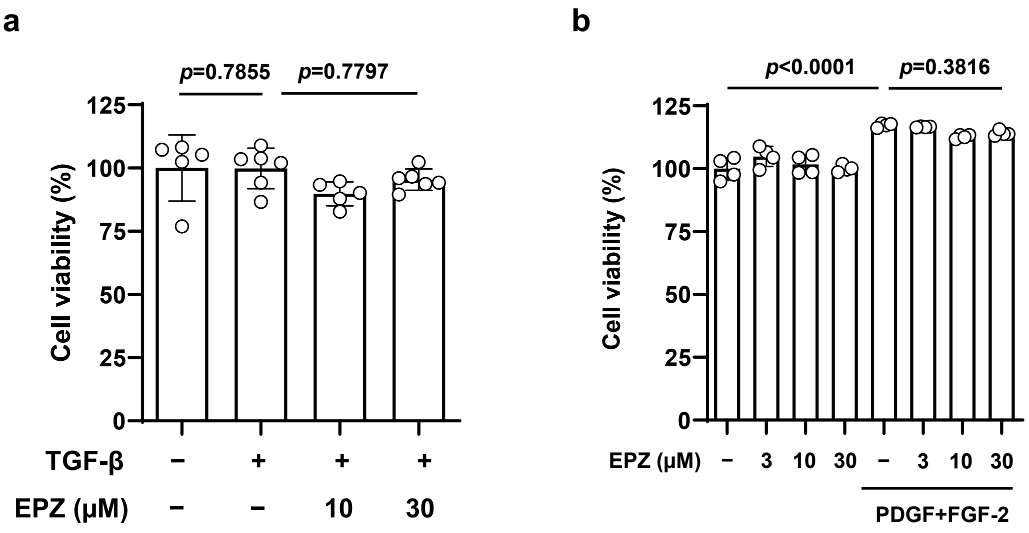

Supplementary Figure 9

Primary cultured human adult cardiac fibroblasts were treated with EPZ015666 at the indicated concentration and then stimulated with or without TGF-β (10 ng/mL) (n=5 biological independent samples) (a) or PDGF+FGF-2 (n=4 biological independent samples) (b). Cell viability was measured after incubation for 48 h. Values are presented as mean ± SD. One-way ANOVA, followed by Dunnett's multiple-comparisons test.

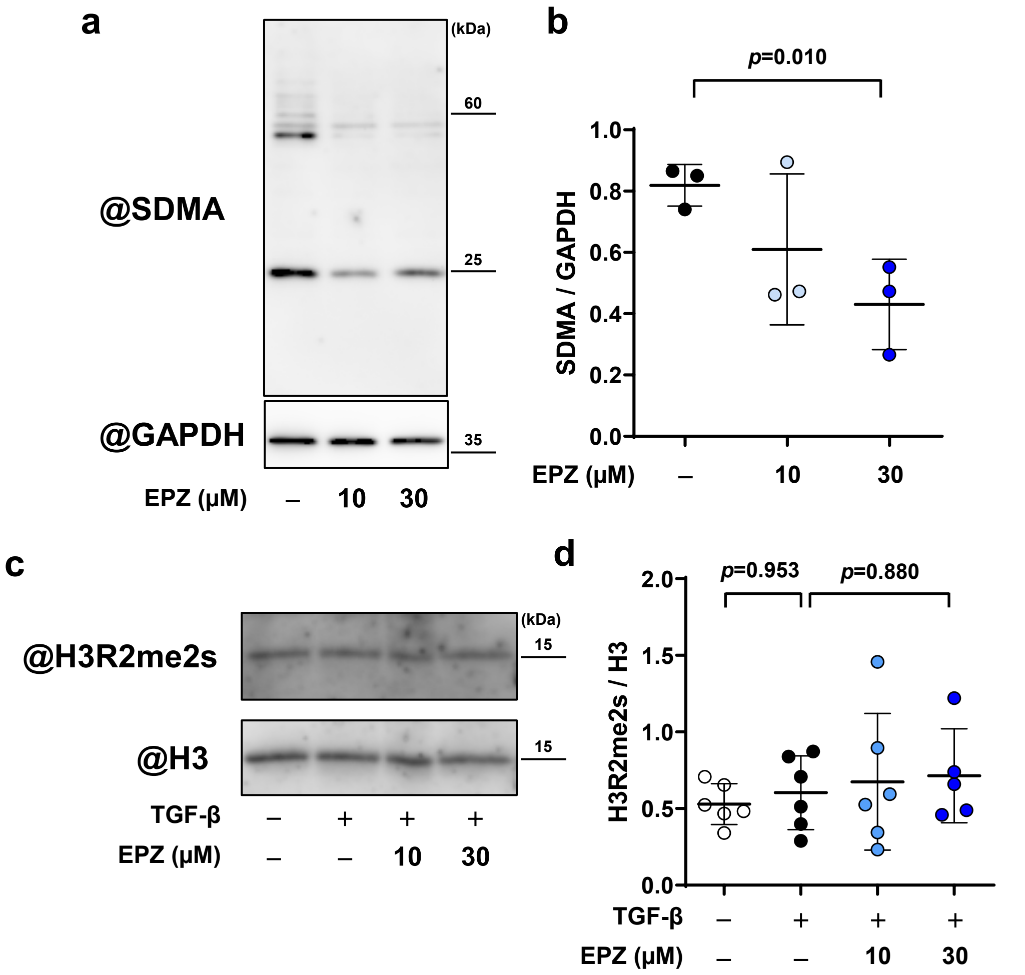

Supplementary Figure 10

Primary cultured cardiac fibroblasts from neonatal rats were stimulated with TGF-β (10 ng/mL) in the presence or absence of the PRMT5 inhibitor EPZ015666 (10 μM or 30 μM). Protein expression levels of SDMA (a, b) and H3R2me2s (c, d) were examined by western blotting. Band density was measured with ImageJ software. Values are presented as mean ± SD. (b) n=3 biological independent samples. (d) n=6 biological independent samples. One-way ANOVA, followed by Dunnett's multiple-comparisons test.

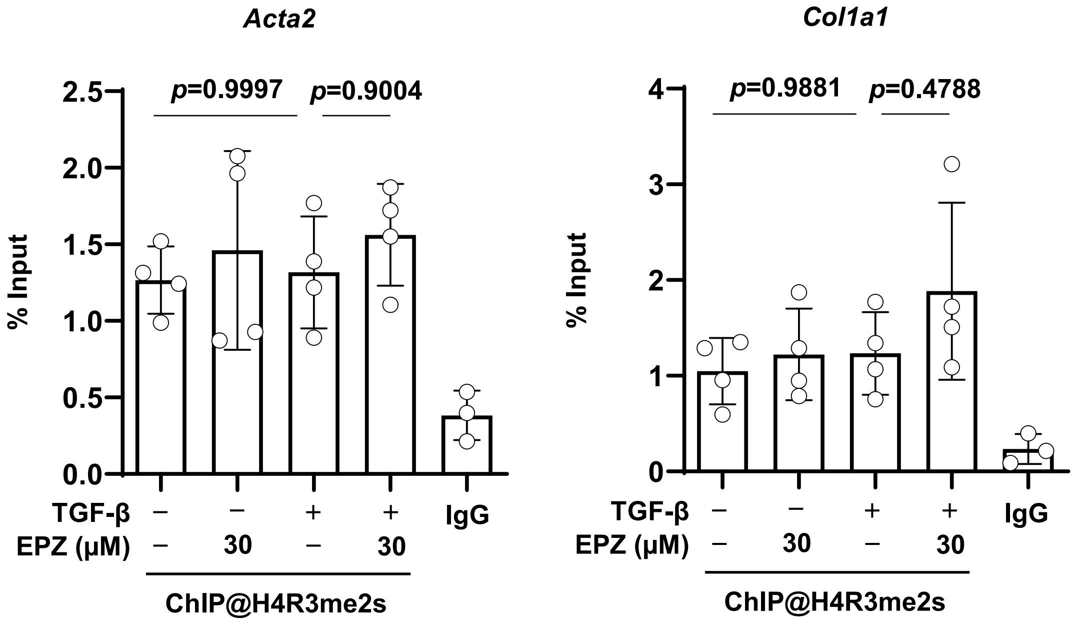

Supplementary Figure 11

The symmetric dimethylation of H4R3 was not increased by TGF-β treatment. Primary cultured cardiac fibroblasts from neonatal rats were stimulated with TGF-β (10 ng/mL) in the presence of EPZ015666 (30 μM) for 6 h. ChIP qPCR assays were performed with anti-H4R3me2s antibody and ChIP primers for *Acta2* and *Col1a1*. Values are presented as mean ± SD. n=4 biological independent samples. One-way ANOVA, followed by Tukey's multiple comparison test.

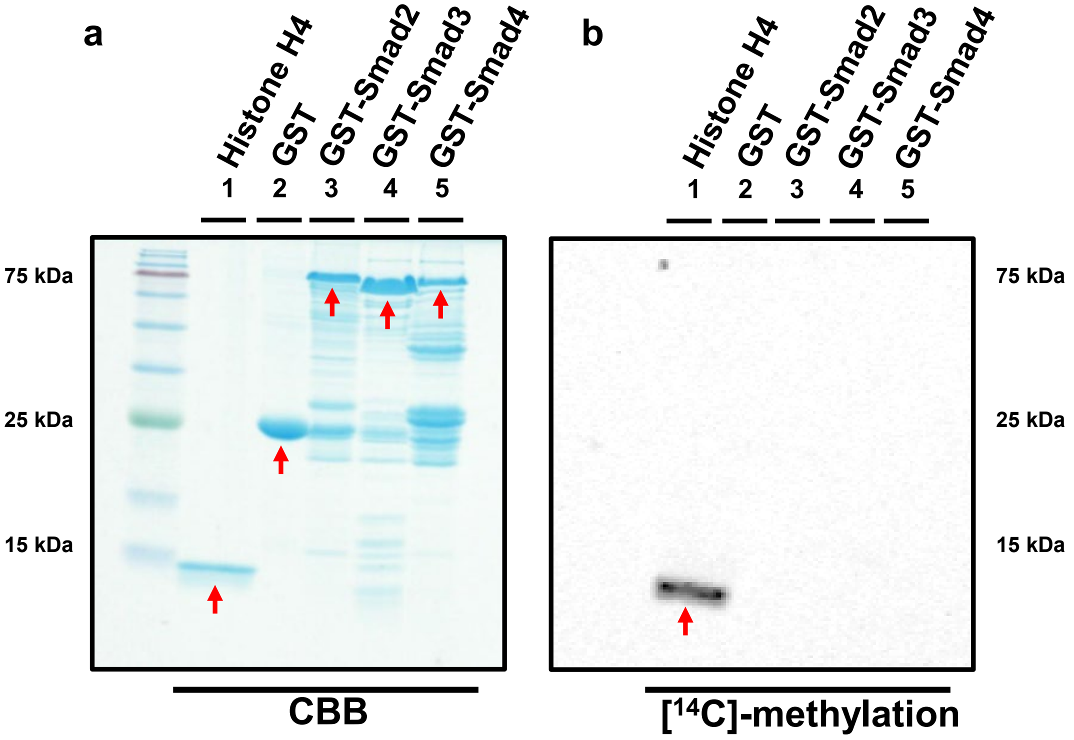

Supplementary Figure 12
PRMT5 does not methylate Smad2, Smad3, or Smad4. Recombinant histone 4 (lane 1) and purified GST-fusion proteins (lanes 2-5) were added to the recombinant Flag-PRMT5/MEP50 complex and [^{14}C]-SAM and then incubated at 30 ºC for 120 min. The samples were separated by SDS-PAGE and stained with CBB after membrane transfer (a). After exposure to the imaging plate, autoradiography was detected with a Typhoon 9400 imager (b). Band positions of each protein and histone 4 are shown by the red arrows. Similar results were obtained twice independent experiments.

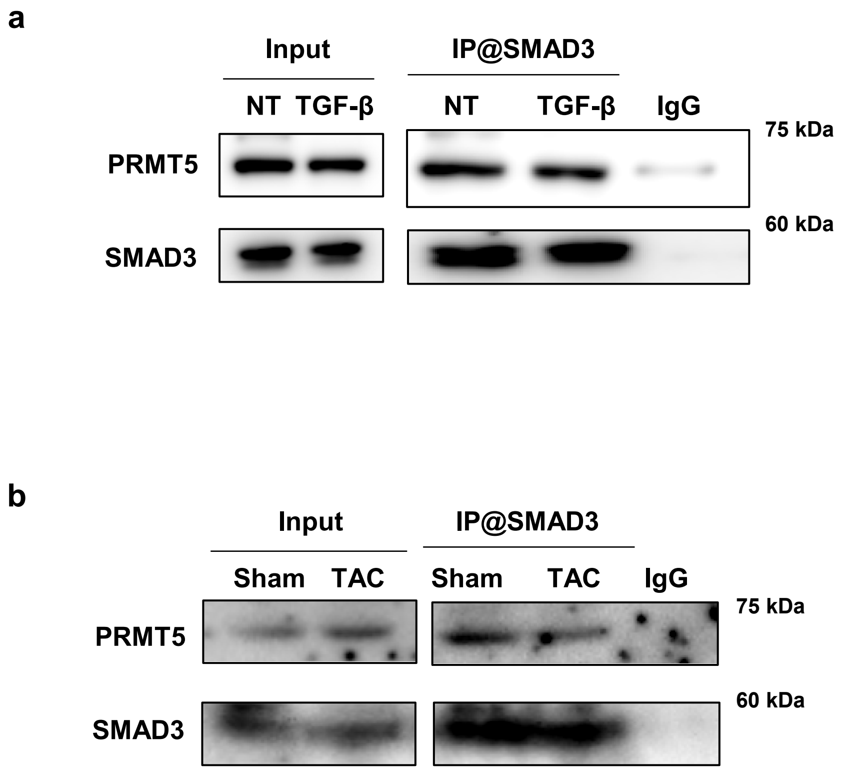

Supplementary Figure 13

Primary cultured cardiac fibroblasts were stimulated with or without TGF-β (a). Transverse aortic constriction (TAC) surgery was performed with male C57BL/6j mice. The mouse hearts with or without TAC surgery were extracted (b). Interaction between Smad3 and PRMT5 in the cells was examined by immunoprecipitation (IP) and western blotting. Similar results were obtained twice independent experiments.

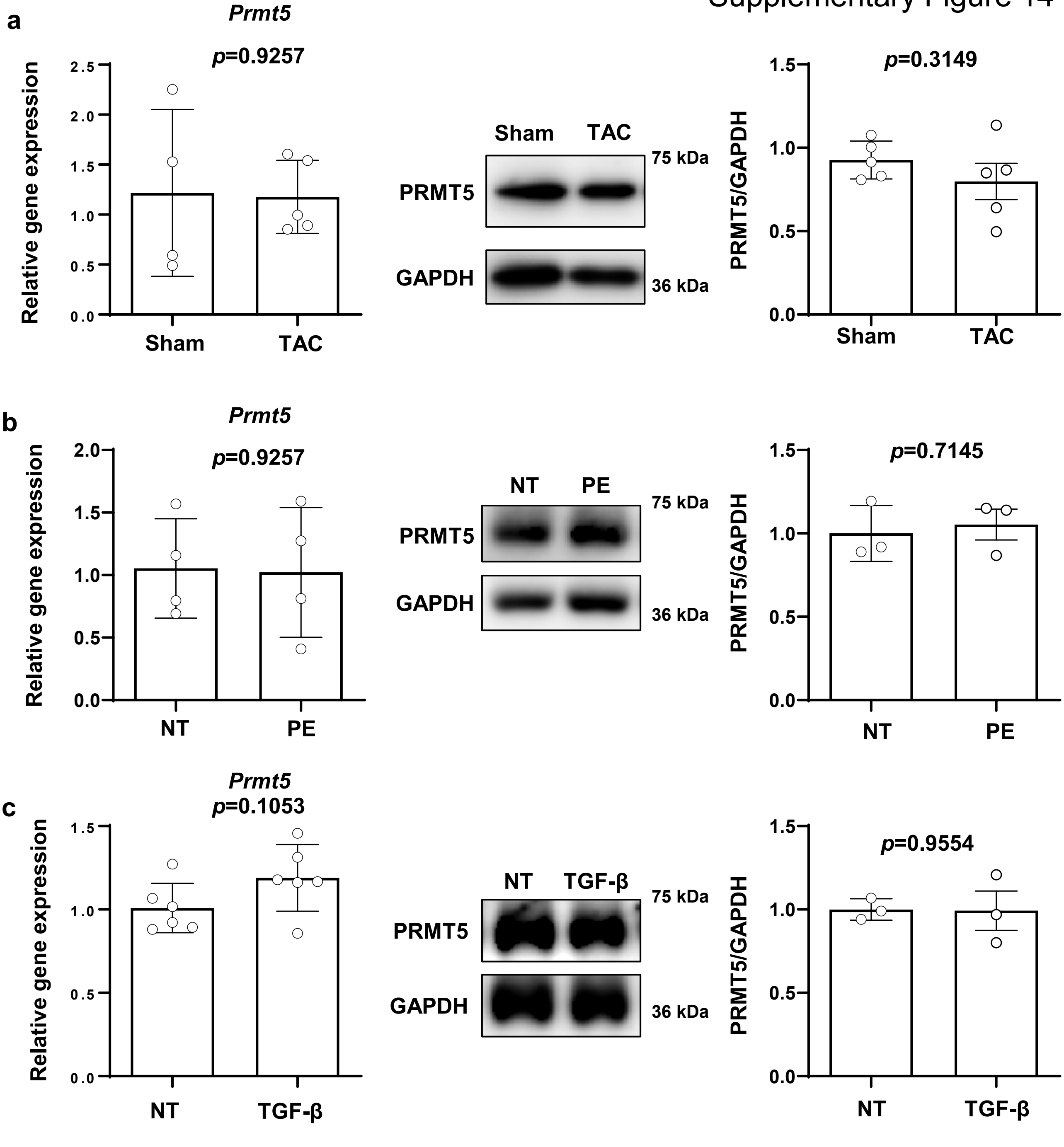

Supplementary Figure 14

The hearts were extracted from mice with or without TAC surgery (n=4 sham and 5 TAC mice (a). Primary cultured cardiac myocytes from neonatal rats were stimulated with or without phenylephrine (PE) (n=4 biological independent samples) (b). Primary cultured cardiac fibroblasts from neonatal rats were stimulated with or without transforming growth factor-beta (TGF-β). (n=6 biological independent samples) (C). PRMT5 expression was examined by qPCR and western blotting. Values are presented as mean ± SD. Unpaired Student's *t*-test.

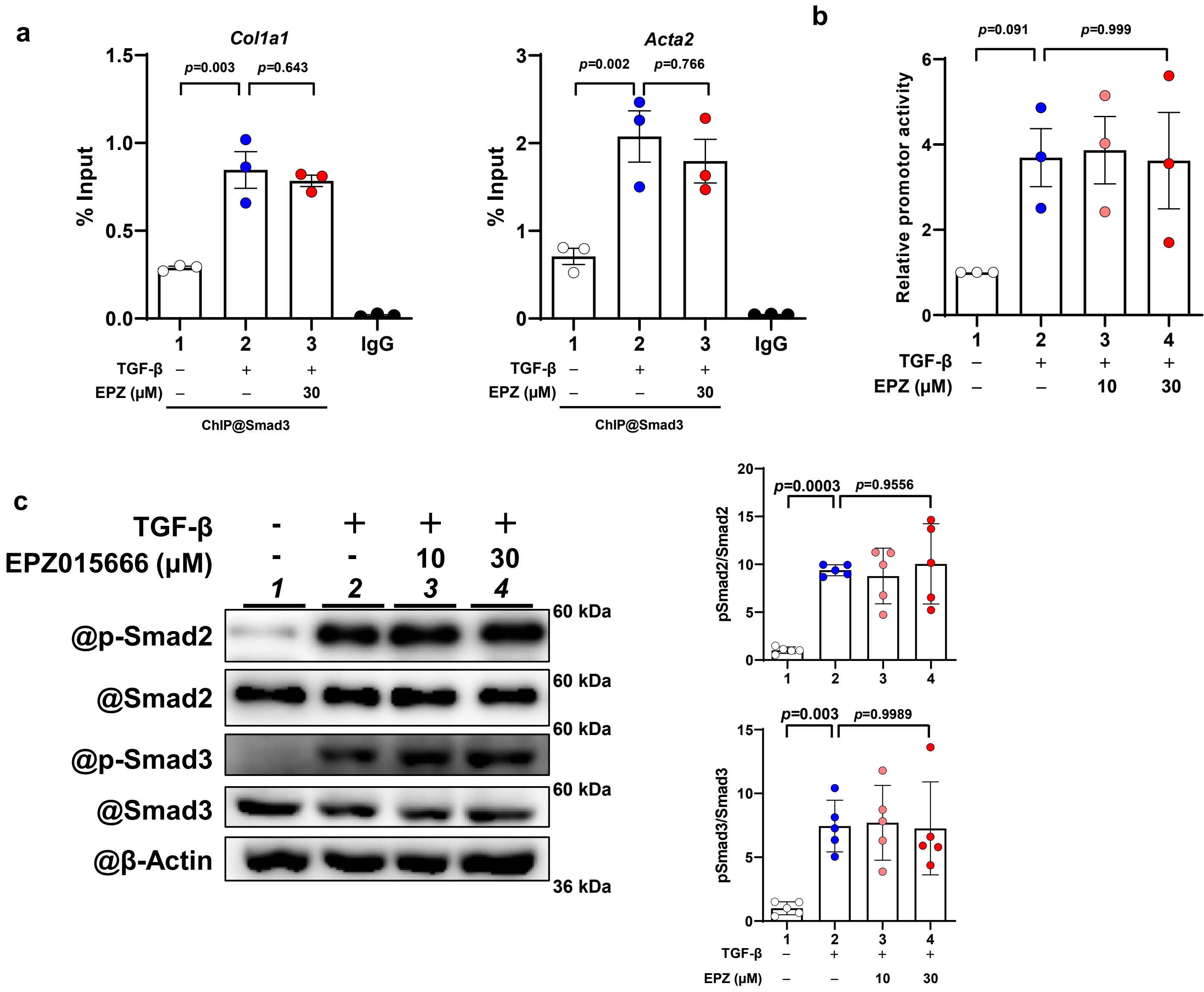

Supplementary Figure 15

The PRMT5 inhibitor EPZ015666 does not suppress activation of the TGF-β/Smad pathway in cardiac fibroblasts. Primary cultured cardiac fibroblasts from neonatal rats were stimulated with TGF-β (10 ng/mL) in the presence of EPZ015666 (30 μM) for 2 h. ChIP qPCR assays were performed with anti-Smad3 antibody and ChIP primers for *Col1A1* and *Acta2*. (n=3 biological independent samples)(a). Primary cultured cardiac fibroblasts were transfected with SBE4-luc and then stimulated with TGF-β (10 ng/mL) in the presence of EPZ015666 for 24 h. The cells were then lysed and transcriptional activity was measured by dual reporter assay (n=3 biological independent samples) (b). Primary cultured cardiac fibroblasts were stimulated with TGF-β in the presence of EPZ015666. Phosphorylation of Smad2/3 was examined by western blotting (n=5 biological independent samples) (c). Values are presented as mean ± SD. One-way ANOVA, followed by Dunnett’s multiple-comparisons test.

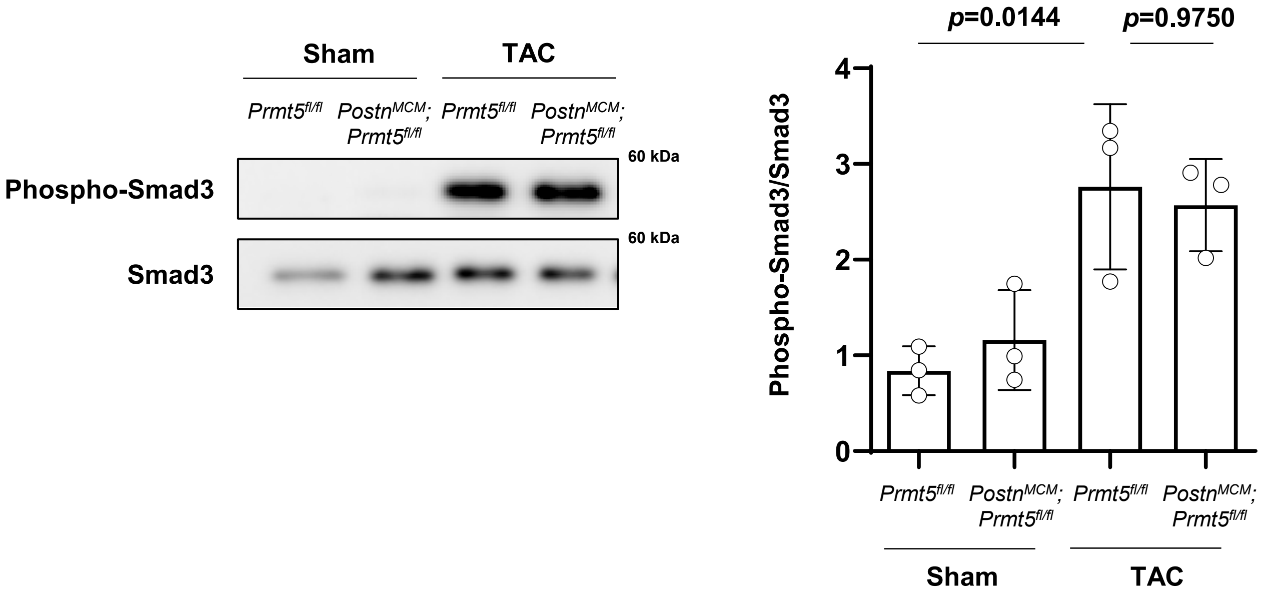

Supplementary Figure 16

PRMT5 knockout in activated fibroblasts does not suppress activation of Smad in hearts. Transverse aortic constriction (TAC) surgery was performed with male *Prmt5*$^{fl/fl}$ and *Postn*$^{MCM;}$*Prmt5*$^{fl/fl}$ mice. The mouse hearts with or without TAC surgery were extracted. Phosphorylation of Smad3 was examined by western blotting. Values are presented as mean ± SD. n=3 mice/group. Two-way ANOVA, followed by Tukey's multiple comparison test. * $p < 0.05$.

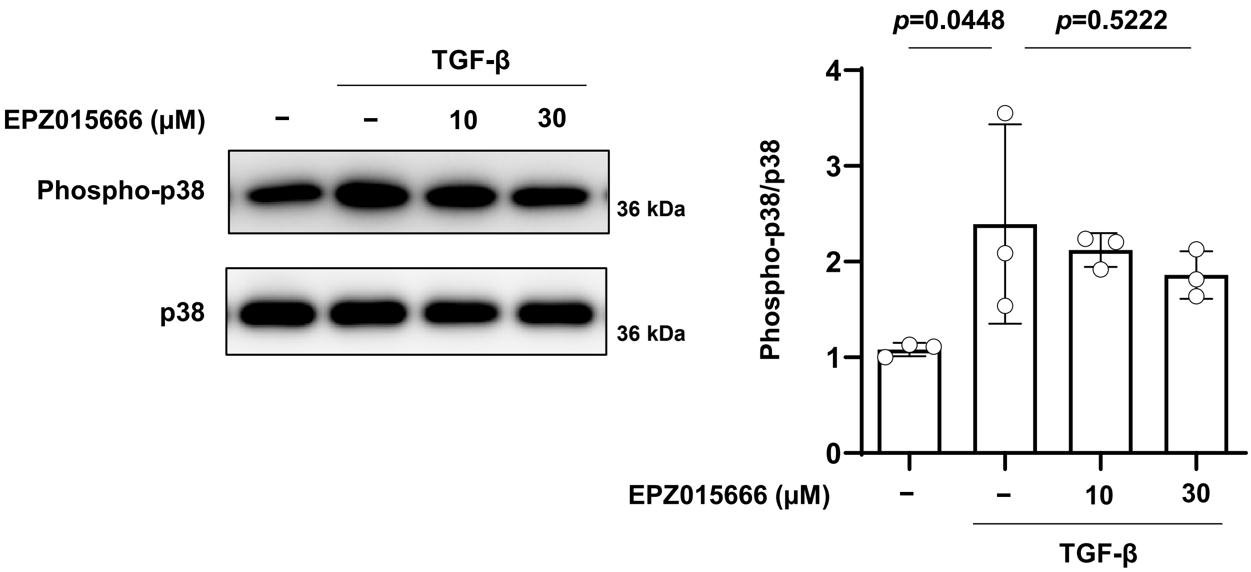

Supplementary Figure 17

PRMT5 inhibitor did not significantly suppress the phosphorylation of p38. Primary cultured cardiac fibroblasts were stimulated with TGF-β in the presence of EPZ015666. Phosphorylation of p38 was examined by western blotting. Values are presented as mean ± SD. n = 3 biological independent samples. One-way ANOVA, followed by Dunnett’s multiple comparison test.

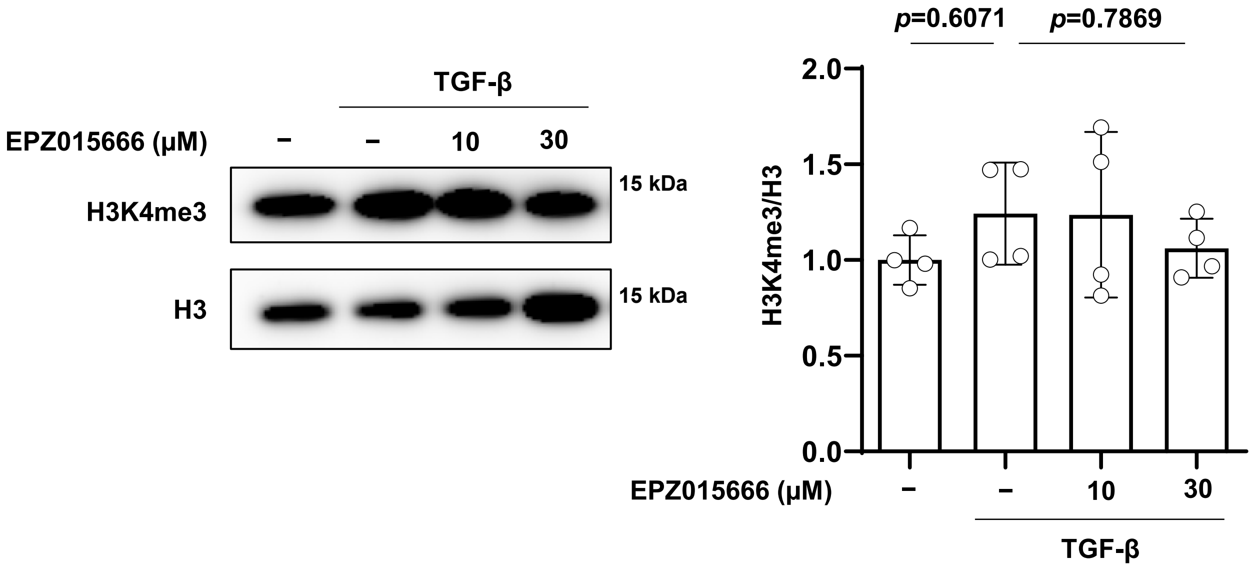

Supplementary Figure 18

PRMT5 inhibitor did not significantly suppress the trimethylation of H3K4. Primary cultured cardiac fibroblasts from neonatal rats were stimulated with TGF-β in the presence of EPZ015666. The trimethylation of H3K4 was examined by western blotting. Values are presented as mean ± SD n=3 biological independent samples. One-way ANOVA, followed by Dunnett's multiple comparison test.

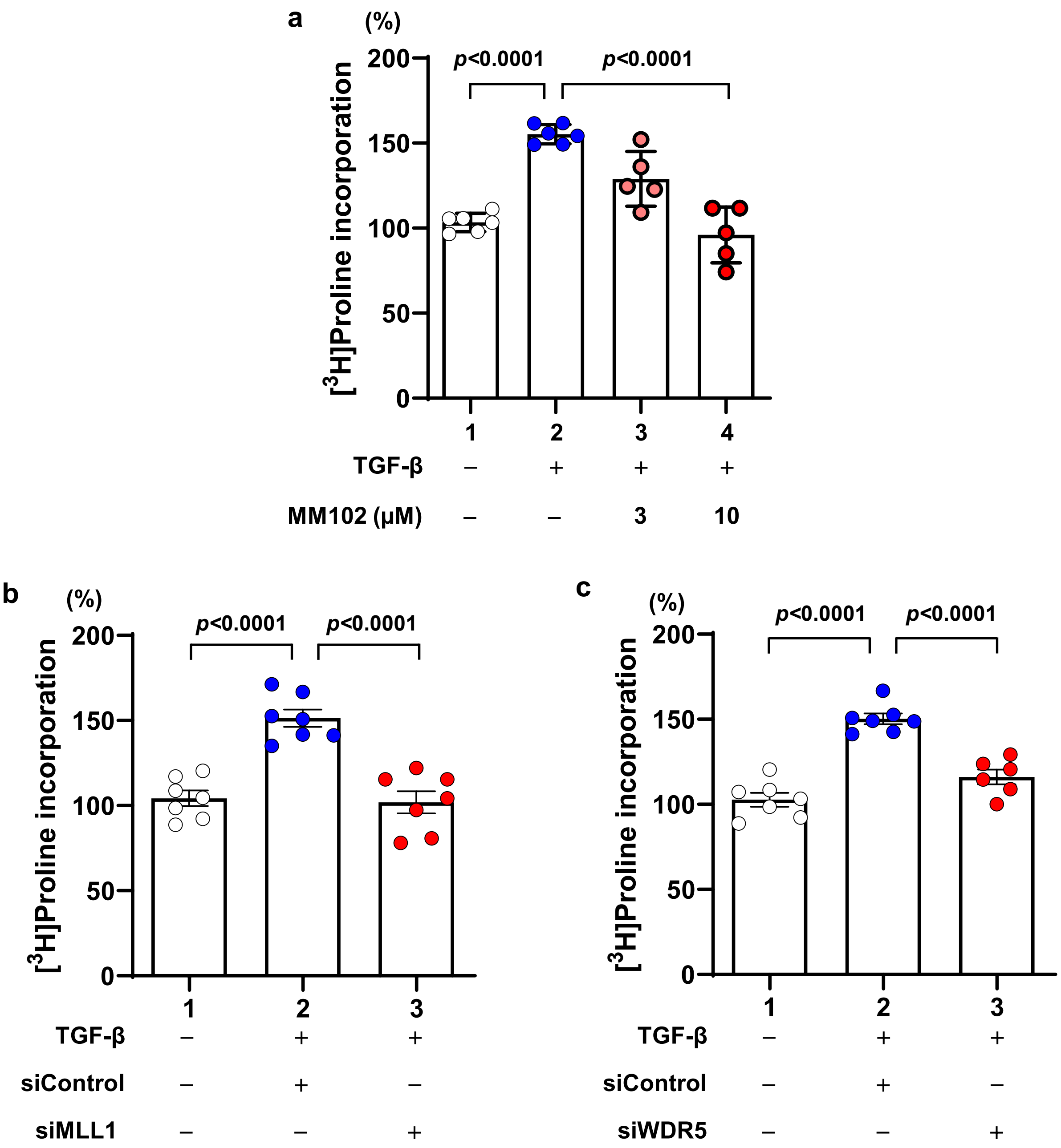

Supplementary Figure 19

Primary cultured cardiac fibroblasts from neonatal rats were treated with the histone methyltransferase inhibitor MM102 at the indicated concentration and then stimulated with or without TGF-β (10 ng/mL) (a). The cells were transfected with siRNA (siControl, siWDR5, or siMLL1) and then stimulated with or without TGF-β (b, c). [^{3}H]-labeled L-proline uptake by the cells was measured after incubation for 48 h. Values are presented as mean ± SD. n=6 (a) and n=7 (b and c) biological independent samples. One-way ANOVA, followed by Dunnett's multiple comparison test.

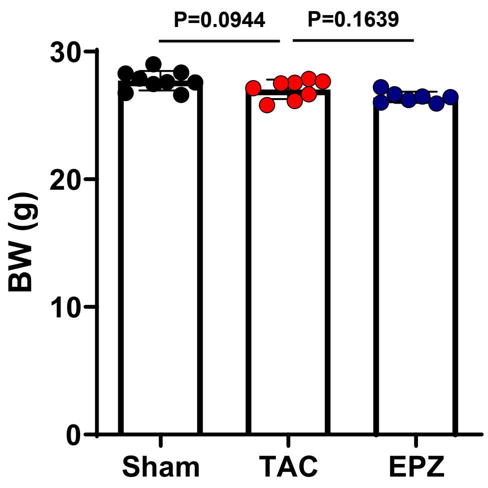

Supplementary Figure 20

EPZ015666 did not exhibit significant body weight change. C57BL/6j male mice (8-10 weeks old) were subjected to TAC or sham operation and then orally administered with EPZ015666 (30 mg/kg/day) or vehicle for 8 weeks. Body weight was measured at 8 weeks after TAC surgery. Values are presented as mean ± SD (n = 9 sham mice, 8 TAC mice, and 7 EPZ mice). One-way ANOVA, followed by Dunnett's multiple comparison test.

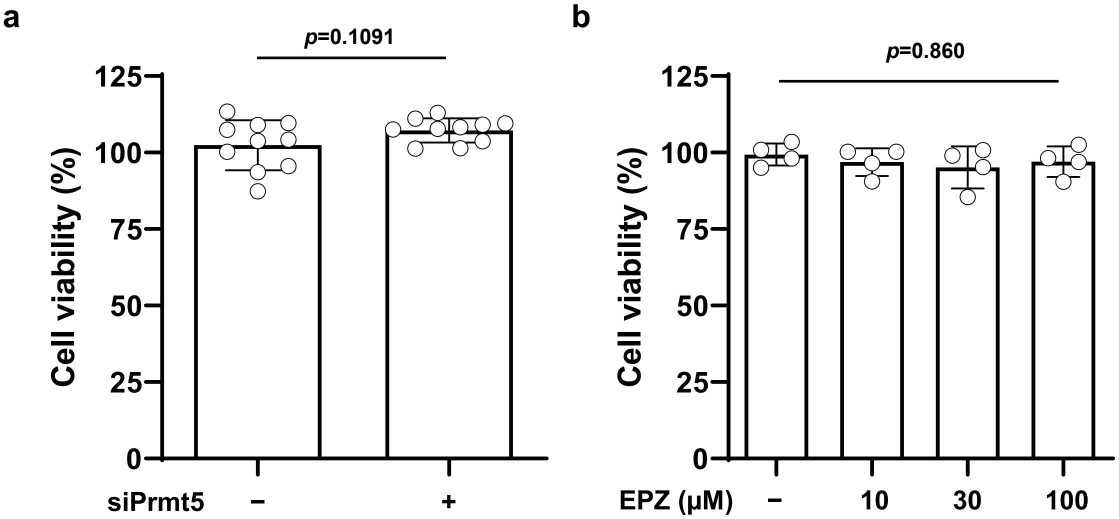

Supplementary Figure 21

(a) Primary cultured cardiomyocytes from neonatal rats were were transfected with siRNA (siControl and siPrmt5) and then incubated for 48 h. Values are presented as mean ± SD (n = 10). Unpaired Student's *t*-test. n=10 biological independent samples. (b) Primary cultured human cardiac fibroblasts were treated with the histone methyltransferase inhibitor EPZ015666 at the indicated concentration. Cell viability was measured after incubation for 48 h. Values are presented as mean ± SD. n=4 biological independent samples. One-way ANOVA, followed by Dunnett's multiple comparison test.

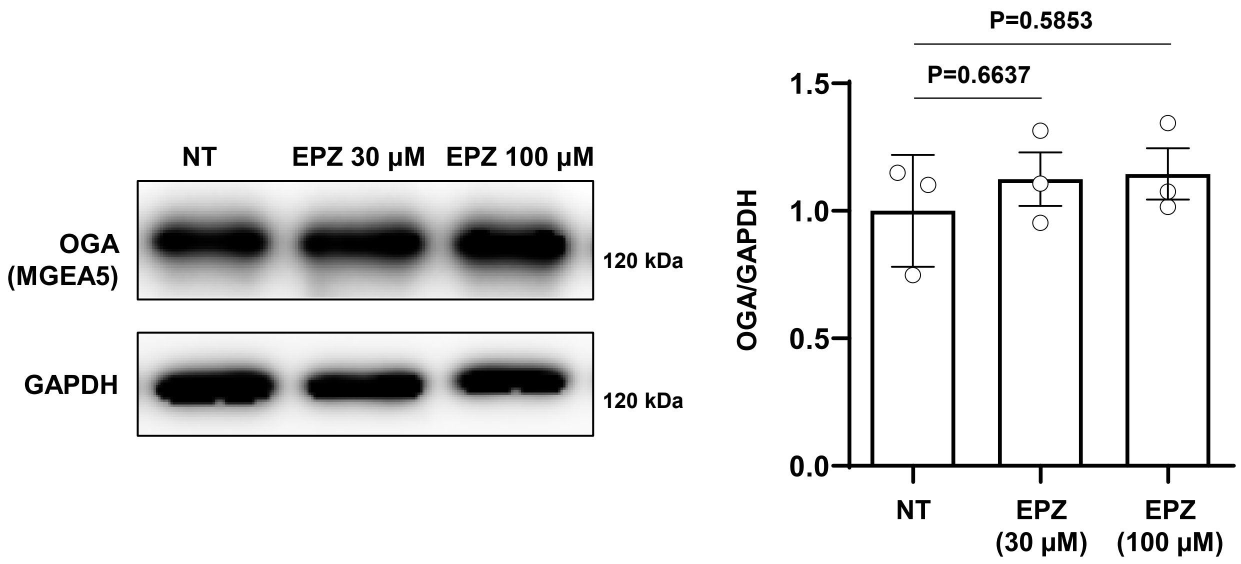

Supplementary Figure 22

PRMT5 inhibitor did not significantly OGA (MEGA5) expression. Primary cultured cardiomyocytes from neonatal rats were treated with EPZ015666. OGA expression was examined by western blotting. Values are presented as mean ± SD. n=3 biological independent samples. One-way ANOVA, followed by Dunnett's multiple comparison test.

Supplementary Table 1
Echocardiography analysis of Prmt5 knockout mice in periostin-positive cells after TAC surgery

	Sham		TAC			
	Prmt5 $^{fl/fl}$	*Postn* ^{MCM} *; Prmt5* $^{fl/fl}$	*Prmt5* $^{fl/fl}$		*Postn* ^{MCM} *; Prmt5* $^{fl/fl}$	
LVIDd, mm	2.6 ± 0.3	2.5 ± 0.2	2.8 ± 0.4		2.2 ± 0.2#	*p* = 0.011
LVPWd, mm	0.8 ± 0.1	0.8 ± 0.1	1.2 ± 0.2***	*p* <0.0001	1.0 ± 0.1#	*p* = 0.028
LVIDs, mm	1.1 ± 0.3	1.1 ± 0.1	1.9 ± 0.4***	*p* <0.0001	1.1 ± 0.3###	*p* = 0.0002
LVPWs, mm	1.2 ± 0.1	1.1 ± 0.1	1.2 ± 0.3		1.1 ± 0.1	
FS, %	56.3 ± 4.7	57.4 ± 4.2	33.7 ± 7.7***	*p* <0.0001	50.9 ± 10.4###	*p* = 0.0004
EF, %	91 ± 2.9	92 ± 2.4	69 ± 8.2***	*p* <0.0001	86 ± 8.4###	*p* <0.0001
LVMI	2.6 ± 0.6	2.5 ± 0.3	4.8 ± 1.1***	*p* <0.0001	2.8 ± 0.6###	*p* <0.0001
Heart rate	524 ± 43	516 ± 47	439 ± 68*	*p* =0.031	455 ± 81	

Data show the mean ± SD.
Two-way ANOVA, followed by Tukey's multiple-comparisons test.
Significant differences are indicated as follows:
* p <0.05, *** p < 0.001 vs Sham-operated *Prmt5* $^{fl/fl}$ group
# p < 0.05, ## p < 0.01, ### p < 0.001 vs TAC-operated *Prmt5* $^{fl/fl}$ group
Exact p value is also shown when there are significant difference.

LVIDd, left ventricular internal dimension at diastolic; LVPWd, left ventricular posterior wall thickness at diastolic; LVIDs, left ventricular internal dimension at systolic; LVPWs, left ventricular posterior wall thickness at systolic; FS, fractional shortening; EF, ejection fraction; LVMI, left ventricular mass index

Supplementary Table 2
Echocardiography analysis of Prmt5 knockout mice in col1a2 positive cells after TAC surgery

	Sham		TAC			
	Prmt5 $^{fl/fl}$	*Col1a2* ^{MCM} *; Prmt5* $^{fl/fl}$	*Prmt5* $^{fl/fl}$		*Col1a2* ^{MCM} *; Prmt5* $^{fl/fl}$	
LVIDd, mm	2.2 ± 0.3	2.3 ± 0.4	2.8 ± 0.3		2.5 ± 0.6	
LVPWd, mm	0.9 ± 0.1	0.9 ± 0.1	1.2 ± 0.2***	*p* =0.0002	1.2 ± 0.1	
LVIDs, mm	0.9 ± 0.1	1.0 ± 0.2	1.8 ± 0.2***	*p* <0.0001	1.4 ± 0.5#	*p* =0.044
LVPWs, mm	1.1 ± 0.1	1.2 ± 0.1	1.3 ± 0.2		1.4 ± 0.3	
FS, %	59.6 ± 4.2	55.2 ± 4.8	34.6 ± 5.2***	*p* <0.0001	47.4 ± 13.0#	*p* =0.024
EF, %	93 ± 2.0	90 ± 2.9	71 ± 6.4***	*p* <0.0001	83 ± 12.8#	*p* =0.029
LVMI	2.3 ± 0.5	2.8 ± 0.7	4.9 ± 1.0***	*p* <0.0001	3.7 ± 1.3	
Heart rate	488 ± 62	434 ± 53	457 ± 79		522 ± 36	

Data show the mean ± SD.
Two-way ANOVA, followed by Tukey's multiple-comparisons test.
Significant differences are indicated as follows:
* p <0.05, *** p < 0.001 vs Sham-operated *Prmt5* $^{fl/fl}$ group
\# p < 0.05, ## p < 0.01, ### p < 0.001 vs TAC-operated *Prmt5* $^{fl/fl}$ group
Exact p value is also shown when there are significant difference.

LVIDd, left ventricular internal dimension at diastolic; LVPWd, left ventricular posterior wall thickness at diastolic; LVIDs, left ventricular internal dimension at systolic; LVPWs, left ventricular posterior wall thickness at systolic; FS, fractional shortening; EF, ejection fraction; LVMI, left ventricular mass index

Supplementary Table 3
Primers used in this study

Name	Experiment	Forward primer (5' to 3')	Reverse primer (5' to 3')
mouse-α-SMA	qPCR	GTCCCAGACATCAGGGAGTAA	TCGGATACTTCAGCGTCAGGA
mouse-Col1a1	qPCR	ACCTCAAGATGTGCCACTC	ACCCTTAGGCCATTGTGTATGC
mouse-Postn	qPCR	ACCTGCAATGACGAAGATCC	TCACTTCTGTCACCGTTTCG
rat-α-SMA	qPCR	GAGCGTGGCTATTCCTTCGTG	CAGTGGCCATCTCATTTTCAAAGT
rat-Col1a1	qPCR	TCAAGATGGTGGCCGTTAC	CTGCGGATGTTCTCAATCTG
18S rRNA	qPCR	CTTAGAGGGACAAGGGCG	GGACATCTAAGGGCATCACA
Col1A1	ChIP	ATCCTTCTGATTTGAGGTC	AGGTGAAACTCCCGTCTG
Acta2	ChIP	AGCCTTCAACTTGTGATCCTC	GGGCAGCATATGAGAATGTG
