# Supplementary material for: Fibroblast-specific PRMT5 deficiency suppresses cardiac fibrosis and left ventricular dysfunction in male mice
Source: Nat Commun. 2024 Mar 19;15:2472. doi: 10.1038/s41467-024-46711-z (PMC10951424; doi:10.1038/s41467-024-46711-z)
Supplement: Supplementary file 3 — Reporting Summary [file 41467_2024_46711_MOESM3_ESM.pdf]

nature portfolio

Corresponding author(s): Yasufumi Katanasaka

Last updated by author(s): Feb 26, 2024

Reporting Summary

Nature Portfolio wishes to improve the reproducibility of the work that we publish. This form provides structure for consistency and transparency in reporting. For further information on Nature Portfolio policies, see our Editorial Policies and the Editorial Policy Checklist.

Statistics

For all statistical analyses, confirm that the following items are present in the figure legend, table legend, main text, or Methods section.

n/a	Confirmed	
☐	☒	The exact sample size (*n*) for each experimental group/condition, given as a discrete number and unit of measurement
☐	☒	A statement on whether measurements were taken from distinct samples or whether the same sample was measured repeatedly
☐	☒	The statistical test(s) used AND whether they are one- or two-sided<br>*Only common tests should be described solely by name; describe more complex techniques in the Methods section.*
☐	☒	A description of all covariates tested
☐	☒	A description of any assumptions or corrections, such as tests of normality and adjustment for multiple comparisons
☐	☒	A full description of the statistical parameters including central tendency (e.g. means) or other basic estimates (e.g. regression coefficient) AND variation (e.g. standard deviation) or associated estimates of uncertainty (e.g. confidence intervals)
☐	☒	For null hypothesis testing, the test statistic (e.g. *F*, *t*, *r*) with confidence intervals, effect sizes, degrees of freedom and *P* value noted<br>*Give P values as exact values whenever suitable.*
☒	☐	For Bayesian analysis, information on the choice of priors and Markov chain Monte Carlo settings
☒	☐	For hierarchical and complex designs, identification of the appropriate level for tests and full reporting of outcomes
☒	☐	Estimates of effect sizes (e.g. Cohen's *d*, Pearson's *r*), indicating how they were calculated

Our web collection on statistics for biologists contains articles on many of the points above.

Software and code

Policy information about availability of computer code

Data collection	No software used.
Data analysis	We used GraphPad Prism 9 software for data analysis.

For manuscripts utilizing custom algorithms or software that are central to the research but not yet described in published literature, software must be made available to editors and reviewers. We strongly encourage code deposition in a community repository (e.g. GitHub). See the Nature Portfolio guidelines for submitting code & software for further information.

Data

Policy information about availability of data

All manuscripts must include a data availability statement. This statement should provide the following information, where applicable:

- Accession codes, unique identifiers, or web links for publicly available datasets
- A description of any restrictions on data availability
- For clinical datasets or third party data, please ensure that the statement adheres to our policy

All data supporting the findings of this study are available within the paper and its Supplementary Information. The data generated in this study are provided in the Source Data file.

Research involving human participants, their data, or biological material

Policy information about studies with human participants or human data. See also policy information about sex, gender (identity/presentation), and sexual orientation and race, ethnicity and racism.

Reporting on sex and gender	N/A
Reporting on race, ethnicity, or other socially relevant groupings	N/A
Population characteristics	N/A
Recruitment	N/A
Ethics oversight	N/A

Note that full information on the approval of the study protocol must also be provided in the manuscript.

Field-specific reporting

Please select the one below that is the best fit for your research. If you are not sure, read the appropriate sections before making your selection.

☒ Life sciences ☐ Behavioural & social sciences ☐ Ecological, evolutionary & environmental sciences

For a reference copy of the document with all sections, see nature.com/documents/nr-reporting-summary-flat.pdf

Life sciences study design

All studies must disclose on these points even when the disclosure is negative.

Sample size	Sample size was determined based on similar studies in Life Science field. Ref: How to calculate sample size in animal studies? J. Charan and N. D. Kantharia, Journal of Pharmacology and Pharmacotherapeutics 2013, Vol. 4, Pages 303-306
Data exclusions	If the value was not detected correctly, the data was excluded.
Replication	Replicate experiments were successful.
Randomization	For in vivo study, mice were randomly assigned to experimental groups. For in vitro study, no formal randomization method was used because it was not relevant.
Blinding	No blinding was performed in this study because it could lead to a risk of mislabeling

Reporting for specific materials, systems and methods

We require information from authors about some types of materials, experimental systems and methods used in many studies. Here, indicate whether each material, system or method listed is relevant to your study. If you are not sure if a list item applies to your research, read the appropriate section before selecting a response.

Materials & experimental systems

n/a	Involved in the study	
☐	☒	Antibodies
☐	☒	Eukaryotic cell lines
☒	☐	Palaeontology and archaeology
☐	☒	Animals and other organisms
☒	☐	Clinical data
☒	☐	Dual use research of concern
☒	☐	Plants

Methods

n/a	Involved in the study	
☒	☐	ChIP-seq
☒	☐	Flow cytometry
☒	☐	MRI-based neuroimaging

Antibodies

Antibodies used	anti-PRMT5 rabbit monoclonal antibody (Merck, 07-405, lot3288011)<br>anti-Smad3 rabbit monoclonal antibody (Cell Signaling Technology, #9523, lot7)<br>anti-WDR5 rabbit monoclonal antibody (Cell Signaling Technology, #13105, lot1)

anti-MLL1 rabbit monoclonal antibody (Cell Signaling Technology, #14197,lot1)
anti-α-SMA mouse monoclonal antibody (Sigma-Aldrich, A5228, lot85382)
anti-HA-tag rabbit monoclonal antibody (MBL Life Science, M132-3, lot015)
anti-FLAG-tag mouse monoclonal antibody (MBL Life Science, M185-3L, lot011)
anti-MGEA5 polyclonal antibody (Proteintech, 14711-1-AP)
Anti-Histone H3 (trimethyl K4) rabbit monoclonal antibody (Abcam, ab8580, lot33417008)
anti-β-actin mouse monoclonal clone AC-15 IgG (Sigma-Aldrich, A1978)
anti-rabbit troponin T antibody (Proteintech, #26592-1-AP)
goat anti-rabbit IgG-HRP (MBL Life Science, 458, lot354)
goat anti-mouse IgG-HRP (MBL Life Science, 330, lot366)
Alexa647 goat anti-rabbit IgG (Thermo Fisher Scientific, Cat #A-21244)
Alexa555 goat anti-mouse IgG (Thermo Fisher Scientific, Cat #A-21429)
Isolectin GS-IB4, Alexa Fluor™ 647 Conjugate (Thermo Fisher Scientific, Cat#I32450)

Validation

All antibodies used in this study are commercially available and validated as follows.
https://www.sigmaaldrich.com/JP/ja/product/mm/07405
https://www.abcam.co.jp/products/primary-antibodies/smad3-antibody-epr19686-chip-grade-ab208182.html
https://www.cellsignal.jp/products/primary-antibodies/wdr5-d9e1i-rabbit-mab/13105
https://www.cellsignal.jp/products/primary-antibodies/mll1-d6g8n-rabbit-mab-carboxy-terminal-antigen/14197?site-search-type=Products&N=4294956287&Ntt=mll1&fromPage=plp
https://www.sigmaaldrich.com/JP/ja/product/sigma/a5228
https://ruo.mbl.co.jp/bio/dtl/A/?pcd=M132-3
https://ruo.mbl.co.jp/bio/dtl/A/?pcd=M185-3L
https://www.ptglab.co.jp/products/MGEA5-Antibody-14711-1-AP.htm
https://www.abcam.co.jp/products/primary-antibodies/histone-h3-tri-methyl-k4-antibody-chip-grade-ab8580.html
https://www.sigmaaldrich.com/JP/ja/product/sigma/a1978
https://www.ptglab.co.jp/products/Cardiac-Troponin-T-Antibody-26592-1-AP.htm
https://ruo.mbl.co.jp/bio/dtl/A/?pcd=458
https://ruo.mbl.co.jp/bio/dtl/A/?pcd=330
https://www.thermofisher.com/antibody/product/Goat-anti-Rabbit-IgG-H-L-Cross-Adsorbed-Secondary-Antibody-Polyclonal/A-21244
https://www.thermofisher.com/antibody/product/Goat-anti-Rabbit-IgG-H-L-Highly-Cross-Adsorbed-Secondary-Antibody-Polyclonal/A-21429

Eukaryotic cell lines

Policy information about cell lines and Sex and Gender in Research

Cell line source(s)

Human cardiac fibroblast and HEK293T cell line are commercially available.
Promocell, https://promocell.com/product/human-cardiac-fibroblasts-hcf/?q=Human+Cardiac+Fibroblasts+%28HCF%29
https://www.atcc.org/products/crl-3216

Authentication

None of the cells used were authenticated

Mycoplasma contamination

Cell lines tested negative for mycoplasma

Commonly misidentified lines
(See ICLAC register)

No commonly misidentified lines were used

Animals and other research organisms

Policy information about studies involving animals; ARRIVE guidelines recommended for reporting animal research, and Sex and Gender in Research

Laboratory animals

C57BL/6 male mice, 8 weeks old, were obtained from Japan SLC, Inc
Col1A2Mer-Cre-Mer (MCM) mice (Stock # 029567) and Periostin (Postn) MCM mice (Stock #029645) were obtained from Jackson Laboratories (Bar Harbor, ME, USA). Male mice (8-10 weeks old) were used in this study.
PRMT5-floxed mice (EMMA ID: 07883) were obtained from Infrafrontier Research Infrastructure (München, Germany). Male mice (8-10 weeks old) were used in this study. All animals were maintained in a pathogen free facility at room temperature 23±1°C in 12:12 h light and dark cycles. The animals were housed in microisolator cages on individually ventilated cage racks filled with aspen chip bedding. The animals were euthanized by cervical dislocation at the end of each animal experiment.

Wild animals

No wild animals were used.

Reporting on sex

Male mice were used in this study based on similar studies in this field.

Field-collected samples

The study did not involve samples collected from the field.

Ethics oversight

Animal studies were reviewed and approved by the Institutional Animal Care and Use Committee of the University of Shizuoka and the National Hospital Organization Kyoto Medical Center.

Note that full information on the approval of the study protocol must also be provided in the manuscript.
